# Some Points in the Choice of a Car

**Published:** 1910-11-12

**Authors:** 


### Motoring Notes.

### SOME POINTS IN THE CHOICE OF A CAR.

The advent of the Olympia Show is the time when the thoughts of many medical men, not only old motorists, but also those who intend forsaking the antiquated gig or dog-cart, turn to the latest thing in motor-cars. There are few things which require more care in selection, and which the ordinary man is less capable of doing unaided. It is one thing to collect the catalogues of a dozen or a score of exhibitors at the Show, take them home, ponder over them and compare points: it is quite another thing, on the information thus collected, to take the definite responsibility of ordering a car which may, for the average medical man, cost anything up to £500. There are many points worthy of careful consideration which should be observed in choosing a car.

First and foremost is the question of price. It need hardly be emphasised that this article is not intended for the man who has an unlimited sum at his disposal, both for the initial purchase and upkeep of his car; it is meant for the average doctor of moderate means, who has a definite limit of price and of yearly cost, and who wishes to secure the best results within that limit. Another point that requires consideration is the use to which the car will mainly be put. While hill-climbing power and speed are the considerations of one, the car for touring, in addition to professional use, must have as its chief characteristics reliability and comfort; and the requirements of the car for purely everyday runabout work are in some respects different again.

Bound up with this question is the vexed one of the number of cylinders, which is a puzzle to all tyros and to not a few who claim to have passed the initiatory stage in motoring. Broadly speaking, the multiplication of cylinders and the increase in horse-power means a proportionate increase in cost. At the same time there are particular instances when a four-cylinder engine can be obtained at a comparatively small additional expense, and when the running expenses are not increased in proportion over the one- or two-cylinder type. As an illustration it might be mentioned that during the present year the writer had a really high-grade 8-h.p. single-cylinder car that consumed more petrol than an equally high-class 10-h.p. four-cylinder car, and was also, of course, more destructive of tyres. There will certainly be a considerable gain in power and efficiency in the higher as against the lower number of cylinders, and while the liability to mishap is also theoretically increased, the risk is in reality infinitesimal in any modern car of repute; the best motor mechanism of to-day is a marvel of efficiency.

For the motorist of small means, or for the man who merely requires a little runabout, cheap to buy and cheap to run, the single-cylinder or the twin will no doubt fill all ordinary requirements. It will not carry a covered body to good purpose, and while it will carry a light four-seated touring body, if put to it, it will be at its best as a two-seater. Naturally, it will not be so successful on steep hills, or with heavy loads, or generally in the work for which higher powered cars are intended. On the other hand, it is easier of manipulation to the tyro, and its greater simplicity will enable him to get a readier grip of car principles than is possible with a high-powered car.

At the other extreme is the high-powered six-cylinder, which is the highest development of the motor magnificent. The arrangement of the cylinders in such a way that the separate impulses overlap gives a wonderful smoothness of movement, which, of course, makes for comfort. The high power gives a masterful sense of something held in reserve for emergencies and sudden spurts; and the chassis can be relied on to carry with ease any description of coach-work. For the average motorist, not to speak of the average medical man, the high-powered six-cylinder must necessarily be but a dream; its first cost and upkeep alike are prohibitive.

And so we come to what is perhaps the most satisfactory car for all-round work, the four-cylinder. The four-cylinder car has a power-stroke at every half-revolution of the crank-shaft, and this is sufficient to ensure evenness of running and the absence of vibration, which are necessary for adequate transmission and comfort of travel. The well-balanced four-cylinder engine works with an efficiency and a silence which leave little room for complaint. As regards the arrangement of the cylinders, it matters little in practice whether they are cast singly, in pairs, or *en bloc*, one of the last two methods being the most usual. As regards horse-power, all ordinary purposes can be served by an engine rated at between 10 and 15 h.p. This is quite sufficient to take a car, with ordinary load, anywhere that a horsed vehicle can go, and to a large number of places that to a horsed vehicle would be impossible.

As to engine details. The valve mechanism is better enclosed, but at the same time ease of access is absolutely imperative. Lubrication should be pump-driven, in order to secure the maximum of efficiency. The Thermo-Syphon system of water circulation is simple and also efficient, provided that the design is right—that is to say, provided the pipes are set at such an angle as will ensure adequate circulation around each cylinder without the possibility of an air-lock. Unless this can be assured, recourse should be had to a centrifugal pump of good dimensions.

The main point of importance in the transmission system is the clutch. The leather-faced cone variety is perhaps still the more popular, although many, the writer included, prefer the plate type. The change-speed mechanism should be on the selective principle, with the gate change, and there should be three speeds forward and a reverse, with the direct drive on the top speed.

*(To be continued.)*

